# Ex-Drug Users' and Health Professionals' Perspectives About School-Based Drug Use Prevention Programs: A Qualitative Study

**DOI:** 10.3389/fpubh.2021.631212

**Published:** 2021-02-12

**Authors:** Teuku Tahlil, Aiyub Aiyub

**Affiliations:** ^1^Department of Community Health Nursing, Nursing Faculty, Universitas Syiah Kuala, Banda Aceh, Indonesia; ^2^Department of Psychiatry, Nursing Faculty, Universitas Syiah Kuala, Banda Aceh, Indonesia

**Keywords:** drug abuse, students, prevention program, healthcare professional, ex-drug users, qualitative study, Indonesia

## Abstract

Adolescents have become a prime target for drug dealers in various countries around the world, including in Indonesia. To reduce the high number of drug users amongst adolescents, effective drug prevention programs should be developed and implemented. The present study aimed to identify effective school-based drug prevention programs for adolescents from the perspectives of former drug users and health professionals. This qualitative research used Focus Group Discussions (FGDs) to collect the data. The study participants consisted of eight Ex-Drug Users (EDUs) and eight Health Professionals (HPs) from health educational institutions and health service settings that were selected through purposive sampling. Data analysis was performed using the qualitative content analysis. Five themes were identified from both EDUs and HPs, including the negative effects of drugs, the socialization of drug abuse, the rehabilitation of drug addicts, the partner collaboration, and the obstacles in preventing drug use. All participants agreed that the drug prevention programs such as school-based drug prevention programs are necessary in order to minimize the adverse effects of drug use. While EDUs tended to focus on the social and psychological effects of drugs, the HPs were more inclined toward the risks of diseases, social impacts, and economic problems of the drugs as the reasons for program importance. In terms of the intervention programs, both EDUs and HPs proposed conducting socialization through the use of active methods and agreed that rehabilitation as an effective way for addict students. Also, both groups of participants agreed on the significance of internal and external support, as well as coordination and collaboration to produce an effective and quality program. While the EDUs cited external factors as the main obstacle to the success of program implementation, the HPs mentioned internal factors of the users. School-based drug prevention programs are an important and potentially effective strategy to reduce the use and the effects of drug use among adolescents. The support of various relevant stakeholders is needed to further increase the effectiveness of the programs and eliminate possible barriers to the implementation of the intervention programs.

## Introduction

Adolescents are at a transitional period in which they try to search for identity and to engage in numerous high-risk behaviors. Adolescents tend to seek pleasure and to be aggressive and moral free; thus, they often encounter conflict psychologically and socially with their families, peer groups, and social environment (1, 2). Psychological and social distress often triggers the occurrence of unhealthy behaviors such as smoking, consuming alcohol, and using drugs (3).

Drugs are dangerous substances. The use of drugs will interfere with the formation and distribution of neurotransmitters and hormone secretion, affecting the growth of the brain area, and nerve circuits (4, 5). Moreover, the use of drugs in adolescents can cause various adverse effects such as low thinking, low academic achievement, high-risk sexual behaviors, increased criminal behaviors, poor interpersonal and social relationships, and increased risk of morbidity and mortality (6, 7).

Despite the harmful effects, drug abuse has become a common problem faced by many countries in the world, including in Indonesia. A survey of the European Monitoring Center for Drugs and Drug Addiction (8) showed that cannabis users in Europe reached 23.5 million people, cocaine 3.5 million people, and opioid 13.5 million people. In Indonesia, the National Narcotics Board (9) reported that the prevalence of drug use in 2017 reached 2.9% for regular users and 9.1% for experimental users, both of which equaled to 1 million (regular users) and 1.8 million (experimental users) of Indonesians. Additionally, the users who had been addicted reached 500,000 people (10). In terms of age, the highest prevalence was at age under 30 years (3%), while in terms of gender, the prevalence of men was greater than women of 12% compared to 4.6% (experimental users) and of 3.7% against 1.7% (regular users), respectively. From the educational background, drug abuse was mostly done by those with low education (9).

Drug abuse among adolescents has been increasingly worrying in Indonesia. Kusumawardhani et al. (11) reported that around 2.5% of 10,736 junior and senior high school students in their survey had used drugs. About 1.1% of the surveyed children started using drugs at the age of seven or younger, and 1.7% consumed drugs from one to more than twenty times during their lifetime (11).

Adolescents are at a critical period for the onset of drug abuse (12). The risk of drug abuse begins to emerge since a child separates from parents and starts entering school. The greatest risk is when an adolescent enters high school. In this period, the adolescent's social condition changes considerably because he/she will face many challenges from social, psychological, and educational aspects. At the age of 15–18 years, adolescents begin to get the trust of parents to regulate themselves, and therefore, they are free to do social activities with peer groups outside their homes (13). This situation will then become the beginning of drug abuse attempts.

Allowing adolescents to be exposed to drugs is similar to letting young people become physically, socially, mentally, and spiritually weak. Drug prevention efforts need to start early, such as between 11 and 12 years (14). During this period, adolescents begin to be exposed to drugs, and usually start using drugs while they are in high school. Therefore, all stakeholders should be fully involved in an effort to eradicate the distribution and use of drugs. Drug prevention is not only the responsibility of the school, but the involvement of all parties, both internal and external, so that a good and drug-free environment can be established. Motivated by this issue, this study was conducted to explore various ideas in a participatory manner from relevant stakeholders. The study was a preliminary research that attempted to identify an effective school-based drug prevention program appropriate for adolescents.

## Methods

### Study Design

This qualitative study used Focus Group Discussions (FGDs) to explore the perspectives of Ex-Drug Users (EDUs) and Health Professionals (HPs) about an effective and appropriate drug prevention program for adolescents in schools. Two FGDs were conducted, included one group of EDUs and one group of HPs. Each FGDs comprised of eight participants.

### Study Participants

Two groups of participants were recruited as samples, namely [1] eight EDUs who have worked as addiction consultants at one of the Provincial National Narcotics Boards, and; [2] eight HPs, comprising health educators (25%), health workers in hospitals (50%) and community health centers (25%) from two districts/cities in Indonesia. The participants were selected by using the purposive sampling technique, taking into account that the selected participants had sufficient knowledge and capacity to provide information in accordance with the objectives of the study. The inclusion criteria of EDUs were: [1] adult participants (20–58 years), [2] have totally quit using drug, [3] have experienced to use rehabilitation unit for recovery, and [4] have experienced as counselor in drug rehabilitation unit. For the HPs, inclusion criteria included: [1] aged between 20 and 60 years, [2] worked as a health worker (services provider or educator), and, [3] have never used drug.

### Data Collection

Data for this qualitative study were obtained through two FGDs (one for EDUs and one for HPs). Semi-structured open ended interview questions related to the implementation of school-based drug prevention programs for adolescents were asked to the participants during each FGDs, including the significance of the program, the appropriate format of the program, the parties needed to be involved in the program, and the use of religious elements in the program. Each FGDs was carried out separately lasting about 60–90 min and recorded using a Voice Recorder. All FGDs were led by the research team and took place at the appointed time and location, as agreed between the research team and participants. The audio recorded of FGDs were transcribed for unit analysis.

### Data Analysis

The data were analyzed by using the qualitative content analysis. The analysis process began by identifying the meaning units of the interview transcripts. The identified meaning units were then inferred in the form of condensed meaning units. Afterwards, the textual and the latent meanings were also identified to determine the themes and the categories relevant with the purpose of the study (15).

## Results

### Characteristics of Participants

The characteristics of participants involved in this study are shown in Table 1. Most study participants were male (68.75%), aged over 35 years (56.25%), married (81.25%), and had above Diploma education (56.25%).

**Table 1:** Characteristics of participants.

**Characteristics**		**Group (f,%)**	
		**Health professionals**	**Ex-drug users**
**Sex**			
	Male	3 (37.50)	8 (100.00)
	Female	5 (62.50)	0 (0)
**Age**			
	25–30	0 (0)	3 (37.50)
	31–35	1 (12.50)	3 (37.50)
	36–40	2 (25.00)	1 (12.50)
	41–45	4 (50.00)	1 (12.50)
	46–50	1 (12.50)	0 (0)
**Marital status**			
	Married	7 (87.50)	6 (75.00)
	Not married	1 (12.50)	2 (25.00)
**Education**			
	Senior high school	0 (0.00)	7 (87.50)
	Diploma III	1 (12.50)	1 (12.50)
	Bachelor/Diploma IV/Profession	4 (50.00)	0 (0)
	Master/Specialist−1	2 (25.00)	0 (0)
	Doctorate/ Specialist−2	1 (12.50)	0 (0)

### Participants' Perception on School-Based Drug Use Prevention Programs

The perceptions of HPs and EDUs on school-based drug use prevention programs are summarized in Table 2. Overall there is no difference in perspective between EDUs and HPs on the implementation of school-based drug abuse prevention programs. There are five main themes suggested, namely (1) The negative effects of drugs, (2) Drug abuse socialization programs, (3) Rehabilitation of addicts, (4) Cooperation partners, and (5) Obstacles to the implementation of drug prevention programs.

**Table 2:** Perceptions of ex-drug users and health professionals on the implementation of school based drug prevention programs.

**Participants**	**Themes**	**Categories**	**Sub-Categories**
Ex-drug users	Negative effects of drugs	Social problems	Users are less empathetic and unruly, commit crime & violate laws.
		Psychological problems	Mind disorders, low concentration, emotional disturbances, and messy lifestyles
	Socialization of drug prevention programs	Media Outreach	Social media, films & posters
		Target groups	Early life and from the beginning of entering school
		Socialization models	Intra and Extracurricular
		Socialization material	Early detection, the dangers of drugs, materials must be in accordance with the target groups, and information conveyed is real and actual and not too vulgar
	Rehabilitation	Rehabilitation models	Outpatient and inpatient rehabilitation specifically for children, and not combined with adults
	Cooperation partners	Internal	Teachers, peer groups, and parents
		External	BNN, Offices of Education and Health, and universities
	Barriers to program implementation	Government support Social support	Financial and legal Stigma and discrimination
Health professionals	Negative effects of drugs	Social problems	Decrease academic achievement and damage social relations (family & community)
		Diseases	Neurological disease, HIV/AIDS and tuberculosis
		Economy	Economic need to obtain substances increases
	Socialization of drug prevention programs	Media outreach	Videos, socio-dramas and advertisements
		Socialization models	Intra and Extracurricular
		Socialization material	Early detection, the dangers of drugs, prohibition of drugs in religion, and information conveyed is not too vulgar
	Rehabilitation	Rehabilitation models	Outpatient and inpatient rehabilitation specifically for children, and not combined with adults
	Cooperation partners	Internal	Students, teachers, and family
		External	Health workers in Puskesmas, hospitals (general & mental), pesantren (Islamic Boarding School) and BNN
	Barriers to program implementation	Government support	Rules and law enforcement
		Users	Low medications awareness, relapse, and substance use suggestions
		Social support	Stigma and discrimination

#### The Negative Effects of Drugs Use

There was a slight difference in perspective between EDUs and HPs on the theme of the negative effects of drugs. The EDUs consider the social and psychological effects of drug users is lack of empathy. One EDU stated as follow:

“*Teenage drug users like to look for fun and don't really care about others.”*

In addition, the EDUs also stated that drug users were difficult to regulate, liked to lie, often committed crimes, making them often legally troubled, as stated by the participants as follows:

“*The signs of drug users include stealing, cheating, lying, and being unruly and stubborn”* “*Teenage drug users often commit crimes to buy drugs”* “*... often violate the law*.”

On the other hand, the HPs considered that social, disease, and economic problems as the negative aspects of drugs. The social problems experienced by drug users included decreased academic achievement and disruption of relationships with family and society. The HPs participants' remarks are shown below:

“*The impact of the use of drugs on adolescents is that [drugs] can disturb the harmonious relationships with parents”*

“*The behaviors of teenage drug users are very disruptive to the community environment*.”

#### Drug Abuse Socialization Programs

The HPs said that socialization media, models, and material should be highly noted. Socialization should guarantee that the students are not so lured that they want to try drugs, as described by the HPs participants below:

“*Socialization can be done through presentations, videos and role play so it can be more interesting”*

“*Socialization in schools must ensure that the students do not want to try to the drugs*.”

According to the EDUs, socialization should be tailored to the target groups, the media, the models, and the material. Their responses are stated in the following:

“*Socialization material needs to be appropriate to the target groups”*

“*Socialization material should not be too vulgar because that can stimulate the teenagers to try the drugs”*

“Consisting of...*early detection*... and. *the dangers of drugs for health and social affairs*.”

In regard with the time of socialization, the EDUs perceived that socialization should begin from when the students enter junior high school, in which the socialization contains actual, real, and worry-free information.

#### Rehabilitation of Addicts

In addition to drug prevention socialization, the EDUs and HPs also consider the medical and social rehabilitation program for the addicts are especially important. Both EDUs and HPs agreed that an outpatient and inpatient rehabilitation model, specifically designated for children/adolescents, need to be provided so that rehabilitation will not cause any negative psychological effects on the children. The participants' statements are shown below:

“*To date, we still do not have a rehabilitation unit specifically for children”* [EDUs].

“*...combining rehabilitation of children with adults can cause psychological pressure for the children.”* [HPs].

The participants also expected that outpatient rehabilitation ensure that addict students can undergo rehabilitation without having their educational process interrupted. The following are one of the EDU participant's responses:

“*If students are identified as drug addicts, they should not be expelled. They can undergo outpatient rehabilitation managed by the national narcotic board*.”

#### Cooperation Partners

In order to ensure the success of drug prevention programs, the participants pointed out the need for coordination and cooperation among related stakeholders. The EDUs and HPs agreed that peer groups, teachers and parents, being important internal parties, should be involved. Their responses are stated below:

“*Schools play an important role in an effort to prevent drug abuse and the role of teachers is very important...”* [HPs].

“*... teachers can become addiction counselors...”* [EDUs].

“*In addition to teachers, peer groups need to be trained so that they can disseminate the prevention of drug abuse.”* [HPs].

There was a different view among the participants on the issue of the external parties. The EDUs perceived that the Indonesia National Narcotics Agency, the Departments of Health and Education, as well as higher educational institutions should be involved, as stated in the following:

“*Cooperation across sectors, such as the Office of Education, National Narcotic Agency, academics and schools are very important*”.

The HPS, on the other side, mentioned that it is necessary to involve the National Narcotics Agency, the center of health services (e.g., community health centers, general and mental hospitals) and the religious elements (Muslim scholars and Islamic boarding schools) in the programs, as stated below:

“*Mental hospital has addiction counselors...”* so “...*[they] can be professional trainers*...”

“*Our people have a strong belief in religious leaders; therefore, their involvement is seen as very important*.”

#### Programs Obstacles

In relation with the obstacles that might be encountered in the implementation of the drug abuse prevention programs, the EDUs considered the government and social support as the obstacles, whereas the HPs added the users as a constraint, in addition to government and social support. Some of their statements are as follows:

“*Families are not open when asked about their children, and some families say their children have migrated even though they are being rehabilitated*... “and “*Suggestion for using drugs is very high, so the relapse cases are also quite high.”*

“*...many important programs previously existed had to stop due to lack of funding.”*

## Discussions

The study aimed to identify effective interventions for school-based drug use prevention programs from the perspectives of EDUs and HPs. When both EDUs and HPs were asked about the importance of drug use prevention programs in schools, the EDUs conveyed that drugs have caused detrimental effects on the growth and development of adolescence period. They had lack of empathy, been difficult to regulate, been highly emotional, and liked to violate and commit crimes, making them often face legal problems during their addiction period of their adolescence. As a result, their lives fell apart and had no future. In addition, the HPs remarked that teenage drug addicts have been often alone and had no harmonious relationships with their families and social environments. The situation happened because these addicts needed a lot of money to buy drugs, and so they often committed theft and other types of crimes. Also, many teenage addicts suffered from chronic illnesses and dropped out of school due to delinquency and inability to adapt academically.

Researchers have investigated potential effects of drug abuse on adolescents. Previous studies (16, 17) have linked drug abuse with school students' low quality of life in mental, psychological, physical, and social dimensions; low of academic performance, absenteeism, school drop-out, getting involve in a crime, low of self-esteem, pleasure seeking, lack of communication skills, and lack of safe recreational space.

Given the magnitude of the destructive effects of drugs, it is crucial to develop effective and quality prevention programs. It is acknowledged (18) that the most essential aspects in improving the quality of a prevention strategy framework are accountability, capacity, and effectiveness. To achieve these, this study attempted to develop a drug prevention model by exploring stakeholder ideas and opinions (EDUs and HPs), and in doing so their participation could be beneficial to produce quality, effective, and acceptable prevention programs.

The FGD results have shown that there are two major themes suggested by the participants for drug abuse prevention programs, including information dissemination of anti-drugs as a preventative measure and rehabilitation of drug addicts as treatment for drug users. Education is the main prevention strategy against drug abuse (19). Dissemination of information about drugs is carried out to increase the target group's knowledge in recognizing substances that are often abused, the consequences of the use of the substances, and to promote anti-drug use attitudes (20).

The results of the FGD further revealed that the EDUs wanted the anti-drug socialization material to be adjusted to the target group (adolescents). In a similar vein, Edalati and Conrod (21) conclude that the drug prevention program material should be designed by taking into consideration the cultural values, the developmental needs, and the attitudes of the target group so that the material will be more effective and relevant to the personality of the target group members. The EDUs also suggested that early detection and the dangers of drugs need to be included in the materials, along with real and actual information. The information should not frighten the target group, but still provide the facts of drugs in real life. The HPs were also in favor of putting early detection and the dangers of drugs in the material. In addition, the HPs considered that a religious perspective on substance abuse needs to be conveyed to increase adolescent beliefs about the dangers of drugs. In terms of the way information is delivered, both EDUs and HPs agreed that the information should not have vulgar content in order to prevent teenagers to try the drugs. One way to disseminate information properly is, according to the HPs, through a field trip to a rehabilitation unit to see the real impact of drug abuse.

Regarding the methods of socialization, the EDUs and HPs also agreed that the use of models, media and material should be adapted to the target group (adolescents). However, the EDUs advocated the use of social media, films, and posters in anti-drug socialization, while the HPs recommended the use of videos, social drama, field trips (rehabilitation units and mental hospitals) and advertisements as the media for disseminating anti-drug information. Buller et al. (22) have written about drug prevention campaigns using mass media and social media for the past 15 years. They assert that the selection of media (broadcasting media, print media, online media, social media, and mobile media) and media content need to be adjusted to the target of the disseminated information. Some media content that can be chosen, according to Botvin and Griffin (20), in the dissemination of information on drug abuse are didactic instruction, discussion, audio/video presentations, displays of substances, posters, pamphlets, and school assembly programs. Further, according to the EDUs, field trips can also provide experience about the dangers of drug abuse, and so these can be considered an effective method for preventing drug abuse behavior among adolescents.

There are three issues targeted by the drug use prevention programs in schools, namely: [1] demand reduction (by giving students skills to say “no to drug”), [2] supply reduction (by crating and developing relevant policies), and [3] reduce the adverse consequences (by treating and referring students with substances abuse to appropriate counseling and treatment (23). The effectiveness of drug use prevention programs in schools are very dependent on the models of anti-drug socialization in schools. In the FGDs, both EDUs and HPs wanted the intra and extracurricular activities to be integrated in the socialization. Both also agreed to insert drug-related material in existing school subjects rather than adding a new subject. The participants stated that drug-related material may be integrated in civics, religion, biology, and physical education. In addition to the intra-curricular model, drug socialization can also be inserted in extracurricular activities such as scouting and school health units.

In the literature review written by Edalati and Conrod (21) of several intervention tests using Randomized Controlled Trials (RCTs) in adolescents and adults, it was found that brief group based intervention sessions (psycho-education, motivational enhancement therapy, and cognitive behavioral therapy) given to high-risk students during school hours were quite successful in reducing the range of drug use to 50% with the effects lasting for up to 3 years. Sloboda and Ringwalt (23) pinpoint that the impacts expected from the dissemination of information about drug use prevention in schools include creating a safe (physical and emotional) and inclusive school environment, promoting positive relationships, and instilling anti-drug norms and behaviors.

Aside of drug use prevention, drug use treatment, and rehabilitation are also the concerns of the participants in the FGDs. When asked about what to do with children who experience drug dependence, the EDUs and HPs agreed that treatment and rehabilitation are important for adolescents who have already been dependent. In the Indonesian Law and Narcotics (24), the Indonesian government incurs severe penalties (maximum death penalty) for drug dealers; however, drug users or addicts, as the victims, are given the opportunity to undergo treatment and rehabilitation programs outside prison (10). Considering the importance of treatment and rehabilitation for drug users (especially adolescents), the EDUs and HPs viewed that a special rehabilitation for adolescents is necessary. The EDUs claimed it is very harmful to combine youth with adults in a rehabilitation unit as this can cause discomfort and stress for the youth. The EDUs also said that outpatient rehabilitation units are better for adolescents whose dependency levels are still low and because inpatient rehabilitation will disrupt their educational process at school. However, in Indonesia, the availability of treatment and rehabilitation units is not yet balanced with drug addicts. In addition, the use of treatment and rehabilitation is also still low. It is therefore necessary to add and improve the quality of treatment and rehabilitation unit services to meet the needs of drug users addiction (10).

Drug abuse prevention programs cannot only be conducted by schools. Thus, the EDUs and HPs suggested the involvement of all stakeholders, both internal and external, to the programs. Both groups agreed that teachers, peer groups, and parents need to be involved as the internal stakeholders in the strategy to prevent drug abuse. There are three parties that need to be involved in the process of preventing and overcoming the victims of drug abuse, such as teachers and parents (internal parties) and health services (external) (21). The involvement of teachers and school counselors as the internal parties is highly required. Some previous studies found that the efforts made by teachers and school counselors were as effective as prevention efforts done by trained clinicians. In addition, the EDUs and HPs also noted the involvement of the National Narcotics Board, the education and health departments, and Islamic boarding schools.

During FGDs, the obstacles to implementing drug use prevention were also discussed. In this case, both groups of participants stated that the government support was not yet optimal. The EDUs further claimed that many prevention and rehabilitation programs had to be canceled due to the lack of funds. Moreover, the legal issue concerned was the lack of legal rules that could ensnare drug dealers. The Indonesian Criminal Code does not specifically regulate drug offenses (25). However, many regulations regarding drug offenses are regulated through the Narcotics Law (No. 35/2009) (24). The opportunity given by the Narcotics Law regarding rehabilitation opportunities for drug addicts is often used by law enforcers to conduct transactions with captured users/dealers. This situation occurs because the standard definition between users and dealers is not yet clear, allowing rise to legal loopholes. In addition to involving the legal rules, the HPs also highlighted the low level of law enforcement in Indonesia, causing the circulation of drug has yet to overcome.

Other than the government support, public support for drug users is also still low. Both EDUs and HPs believed that stigma and discrimination against drug users was still very high within the Indonesian society. Stigma has been very high in society (26). Families cannot trust drug users and health care workers provide poor care to the users. This has an effect on the low help-seeking behavior of drug users, leading to low participation in treatment and rehabilitation. Sabarinah (10) found that the rate of drug users utilizing treatment and rehabilitation (TR) in Indonesia was about 15%. While this figure was not merely affected by the stigma, the number and quality of services from TR were indeed a problem.

The main limitation of the current study is the findings were merely based on data obtained from FGDs with EDUs and HPs. The findings may not represent the need of adolescents. Thus, further studies which include students and other relevant stakeholders are required to further explore the most appropriate and effective school-based drug use prevention programs for adolescents.

## Conclusion and Suggestions

Drug prevention programs among junior high school students are considered highly significant in order to prevent the adverse effects of drug abuse. The results showed that in addition to the prevention program, treatment, and rehabilitation should also be carried out to cure addict students. In the process of treatment and rehabilitation, the aspects of education and psychology should always be considered. Therefore, special rehabilitation of children and adolescents is needed to minimize their psychological stress compared to when treated together with adults. In addition, outpatient rehabilitation units should also be developed so that the addict students can undergo treatment and rehabilitation without having to leave school.

Drug prevention programs are not only the responsibility of schools, and thus, internal and external coordination and cooperation are necessary to produce an effective and quality program. Support from various relevant stakeholders will also be needed to help improve the effectiveness of drug abuse prevention programs.

## Data Availability Statement

The datasets used in the current study are available from the corresponding author on a reasonable request.

## Ethics Statement

The studies involving human participants were reviewed and approved by Research Ethics Committee of the Nursing Faculty of Universitas Syiah Kuala, Banda Aceh, Indonesia. The patients/participants provided their written informed consent to participate in this study.

## Author Contributions

TT wrote a proposal for funding, led the study design and data collection, and assisted in data analysis and writing of the manuscript. AA assisted in data collection, analyzed the data, and wrote the manuscript.

## Informed Consent

Information about the study was delivered orally and in writing to all study participants. All participants provided their written consent for their involvement in this study.

## Conflict of Interest

The authors declare that the research was conducted in the absence of any commercial or financial relationships that could be construed as a potential conflict of interest.
